# Thyroid paraganglioma in a patient with a history of carotid and vagal paraganglioma: metastatic or primary tumor?

**DOI:** 10.1093/jscr/rjab102

**Published:** 2021-04-22

**Authors:** Natesh Yepuri, Gautam R Vanga, Rana Naous, Sudhakar Kinthala

**Affiliations:** 1 Department of Surgery, SUNY Upstate Medical University, Syracuse, NY 13210, USA; 2 Department of Pathology, SUNY Upstate Medical University, Syracuse, NY 13210, USA; 3 Department of Anesthesiology, Robert Packer Hospital, Sayre, PA 18840, USA

**Keywords:** Paraganglioma, Thyroid, Familial, Metastatic, Primary, Succinate dehydrogenase (SDH) genes

## Abstract

Paragangliomas (PGs) are extremely rare multicentric neoplasms. Hereditary or familial PGs are associated with germline mutations in succinate dehydrogenase genes, seen in one-third of cases. Primary PGs of the thyroid are uncommon neuroendocrine neoplasms that account for 0.012% of all head and neck lesions. Although majority of these tumors are solitary, familial PGs are associated with synchronous tumors (carotid/vagal). We report an interesting case of primary thyroid PG in a patient with a previous history of a right carotid body, right vagal PGs and positive familial history, confining the differential diagnosis to recurrent lesions, which is the most common occurrence or new primary or a metastatic lesion. However, long interval and surgical anatomy suggests the diagnosis to be a primary lesion. In conclusion, although these lesions present multicentrically present at varying intervals, their occurrence at anatomically distinct sites should raise the concern for a new primary PG.

## INTRODUCTION

Paragangliomas (PGs) are rare neoplasms and multicentric ones that are usually associated with familial syndromes. Hereditary or familial PGs are usually associated with germline mutations in succinate dehydrogenase (SDH) genes and are seen in one-third of PG cases, whereby tumors are multiple, more commonly malignant, and have an increased association with pheochromocytoma. PG of the thyroid is an exceedingly rare neuroendocrine tumor that accounts for 0.012% of all head and neck tumors. The majority of thyroid PGs are solitary lesions, with only very few cases reported to present with synchronous carotid body tumors [1, 2]. Although familial PGs are associated with synchronous PGs in the neck (carotid/vagal), the occurrence of these PGs at different time periods after resection at close anatomical sites raises the concern for metastatic lesions.

It has been hypothesized that thyroid PGs embryonically either originate within the thyroid capsule or displaced inferiorly to the lateral side of the thyroid [3]. We report an interesting case of primary thyroid PG presented after right carotid body tumor resection 13 years ago and a right vagal PG resection 24 years ago, and with a positive familial history raising the index of suspicion for a metastatic tumor. The presence of a previous history of malignancy and a new PG lesion confines the differential diagnosis to recurrent neoplasm from the previous site, which is the most common occurrence or a new primary or a metastatic lesion. However, in the present case, the long interval after surgical resection and surgical anatomy indicates this lesion is most likely a ‘Primary’ thyroid neoplasm.

## CASE PRESENTATION

**Figure 1:**
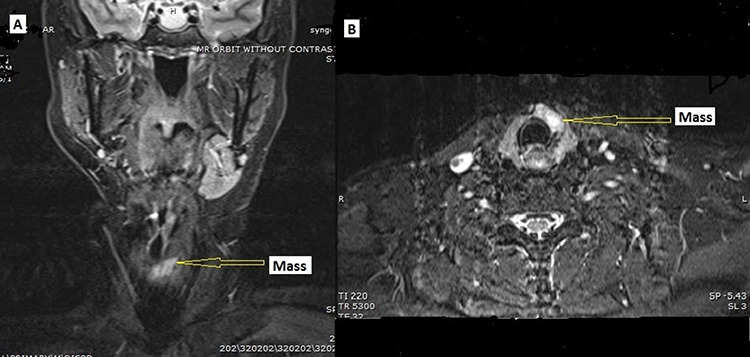
MRI. (**A**) Coronal and (**B**) axial, STIR image demonstrating hyperintense mass lesion (arrow pointing) within the isthmus extending into the left thyroid lobe.

A 58 y/o F with a history of a right carotid body tumor 13 years ago, and a right vagal PG 24 years ago status post excision presented with a thyroid nodule. The patient family history is significant for carotid body tumor in her brother; physical examination was unremarkable with no palpable neck masses or lymph nodes except for nodule. Serum TSH, FT3 and FT4 were consistent with euthyroid state, and serum calcitonin was normal. Thyroid ultrasonography showed a dominant solid hypoechoic nodule in the isthmus extending into the left lobe (24 × 8 × 23 mm), with associated margin and increased central and peripheral vascularity w/o calcification. Further workup with magnetic resonance imaging (MRI) demonstrated hyperintense mass lesion within the isthmus extending into the left thyroid lobe (Fig. 1A and B). CT scan of the chest, abdomen and pelvis were negative. Fine needle aspiration biopsy of the nodule revealed atypia of undetermined significance.

**Figure 2:**
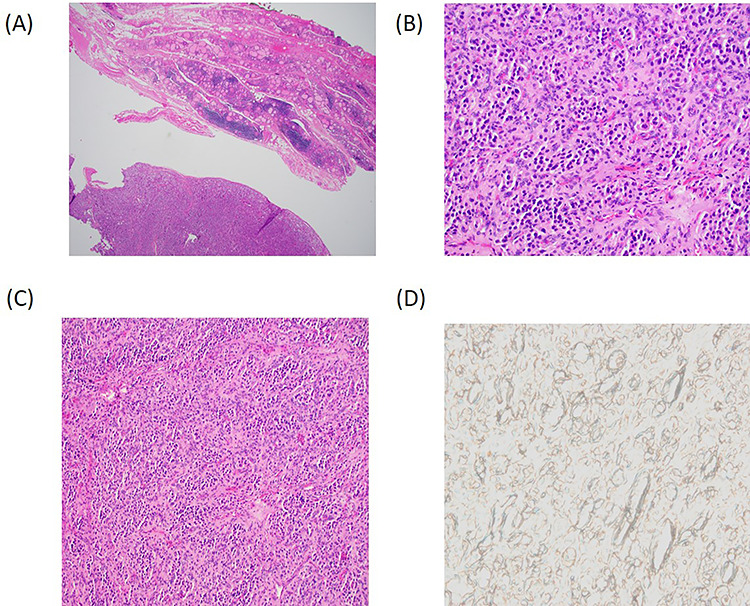
Histopathology. (**A**) Low power magnification depicting a vaguely lobular proliferation of round cells adjacent to benign thyroid parenchyma. (H&E, 4×). (**B**) Higher power magnification demonstrating the nested arrangement of PG with a round to oval cells and eosinophilic cytoplasm set within a highly vascularized stroma. (H&E, 20×). (**C**) Zellballen pattern; a characteristic feature of PG (H&E, 10×). (**D**) Negative SDH-B immunostain suggests the familial or hereditary nature of the PG.

With consideration of the patient’s age and the suspicion of thyroid malignancy, a total thyroidectomy was performed without complications, and there were no signs of local tissue invasion or abnormal cervical lymphadenopathy. Definitive histopathology (Fig. 2A–C) and immunohistochemistry were consistent with feature of PG. SDH-B immunostain was negative in the neoplastic cells (Fig. 2D). The final pathology was consistent with primary thyroid PG. To assess for the presence of synchronous or metachronous hormonally active neuroendocrine tumors, urinary metanephrine and nor-metanephrine excretion was tested at 1-, 2- and 3-years follow-up. There is no evidence of local recurrence at neck ultrasound or distant staging at 5-year follow-up after surgery.

## DISCUSSION

Head and neck PGs (HNPGs) represent the diffuse neuroendocrine system that is parasympathetically derived and rarely secrete catecholamines (non-functional). Carotid body tumors (50–70%), Jugular PG (20–40%) and Vagal PG (5%) constitute the majority of HNPGs. However, primary thyroid PGs are extremely rare [4, 5].

Thyroid PGs are often present in the fourth or sixth decade of life with a female predominance. In a recent systematic review of 46 cases by Navaratne *et al*. [6], it is reported that these tumors present as a slowly enlarging neck mass, with only very few cases presenting with hypertension. In a patient with suspected PG, head and neck imaging with contrast-enhanced CT or MRI is the preferred diagnostic modality. It can help identify the multicentric lesions, delineates the anatomic distribution of the mass and its relationship with other vascular structures. In most cases, FNA is non-diagnostic.

Surgical resection is the mainstay of treatment. Post-operatively, the diagnosis is confirmed microscopically by the presence of chief cells and sustentacular cells. The chief cells form a nested proliferation ‘Zellballen’ appearance characteristic of PG [7]. Despite few cases reporting local extension into surrounding structures, these tumors are generally considered benign, with no metastasis reported after resection. Due to the rarity of these tumors, there is a lack of clear understanding of their clinical behavior and natural history; hence, postoperative hormonal evaluation for any functional disease, imaging evaluation for multicentric and metastatic disease, and genetic counseling is recommended.

Nearly one-third of PGs have a genetic predisposition and are more often multiple, commonly malignant, and associated with other tumors, including pheochromocytoma. Sporadic HNPGs, on the other hand, are rarely malignant (4–16%). Hereditary PGs generally present with mutations or double hit inactivation of SDH genes, leading to dysfunction of mitochondrial complex II and analysis of SDH mutations (*SDHA, SDHB, SDHC* and *SDHD*) aids in the diagnosis and screening of familial PG syndrome [8, 9]. In our case, SDHB staining was negative; therefore, the tumor was most likely familial.

## METASTATIC OR PRIMARY

In our case, given the previous history of carotid, and vagal tumors, any mass in the thyroid would raise the question of a recurrent lesion that metastasized to the thyroid since the vagal nerve runs close to the carotid bifurcation. However, long interval, radiologic findings, surgical anatomy and definitive histopathology confirms the lesion to a ‘primary’ thyroid neoplasm. Therefore, careful evaluation of the anatomical relationship of the mass to the carotid bifurcation/carotid body region by imaging allows for this distinction.

## CONCLUSIONS

Thyroid PGs are extremely rare tumors. Although familial syndromes present with multiple synchronous HNPGs, since these are slow-growing tumors, they can present after a very long interval at different anatomical sites, even after resection. Therefore, along with the search for other synchronous or metachronous tumors, any tumor mass in the nearby structures should raise the concern for a new primary PG.

## CONFLICT OF INTEREST STATEMENT

None declared.

## FUNDING

None.
